# Features of attachment in women with eating disorders

**DOI:** 10.1192/j.eurpsy.2024.416

**Published:** 2024-08-27

**Authors:** M. N. Samoilova, N. D. Semenova

**Affiliations:** ^1^Institute of Clinical Psychology and Social Work, Pirogov Russian National Research Medical University; ^2^Moscow Research Institute of Psychiatry – a branch of the V. Serbsky National Medical Research Centre for Psychiatry and Narcology of the Ministry of Health of the Russian Federation, Moscow, Russian Federation

## Abstract

**Introduction:**

Eating disorders (ED), especially anorexia nervosa, are known to be the most associated with high mortality rates among psychiatric conditions. In many cases, they are resistant to treatment because patients tend to show low compliance, concealing symptoms from doctors. Body image concerns may affect communication and hinder building connections with people, making patients feel alienated.

**Objectives:**

The study aimed to examine the specific characteristics of attachment styles and evaluate their interrelationships with psychological features in women with eating disorders.

**Methods:**

A total of 52 women with a clinical diagnosis of eating disorder (namely, 26 with anorexia nervosa (AN) and 26 with bulimia nervosa (BN)) and 43 healthy controls were included in the study. All participants completed the following psychometric scales: Relationship Questionnaire (RQ), Experience in Close Relationships (ECR), Relationship Profile Test (RPT), and Multidimensional Perfectionism Scale (MPS). The Kolmogorov-Smirnov normality test was applied, confirming a non-normal distribution of the sample; therefore, the non-parametric Mann-Whitney test and Spearman statistics were administered.

**Results:**

The results show a marked difference between the two groups. In the ED patients’ group, only 15% of respondents classified their attachment style as secure, compared to 37% of the participants in the control group. 85% of women in the ED group identified themselves as having one of the insecure attachment styles (anxious, avoidant, or disorganized). The level of relationship anxiety and the rate of relationship avoidance in the respondents of the ED group is 27% and 19% higher, respectively, compared to the control group. Likewise, the level of destructive interpersonal overdependence is 20% higher in the ED patients’ group respondents, whereas healthy dependence is 18% lower compared to the controls. The respondents with ED showed 18% higher self-oriented perfectionism and 39% higher socially prescribed perfectionism. A direct correlation between avoidant attachment style and destructive interpersonal overdependence was found in women with diagnoses belonging to the ED group. When comparing AN and BN patients, no statistically significant differences in the distribution and peculiarities of attachment styles in the anorexia and bulimia groups were found.

**Conclusions:**

The study has proved the hypothesis that insecure attachment is more common among women with eating disorders than those without the diagnosis. We suggest a more profound scientific elaboration of the attachment in ED patients to increase the level of compliance of this group of patients, to improve the effectiveness and reduce the duration of treatment, and develop new therapeutic approaches to cure this disease.

**Disclosure of Interest:**

None Declared

